# New-onset hyperglycaemia and prolonged systemic corticosteroids therapy in mild COVID-19 patients as major risk factors for invasive mucormycosis: a preliminary study 

**DOI:** 10.18502/CMM.7.3.7254

**Published:** 2021-09

**Authors:** Satyendra Khichar, Subhashree Samantaray, Deepak Kumar, Veena Mobarsa, Vidhi Jain, Vidhu Sharma, Kapil Soni, Bikram Choudhury, Amit Goyal, Durga Shankar Meena, Srikanth Srinivasan, Naveen Dutt, Pankaj Bhardwaj, Ashwini Agarwal, Mahendra Kumar Garg, Sanjeev Misra

**Affiliations:** 1 Department of Internal Medicine, All India Institute of Medical Sciences, Jodhpur, India; 2 Infectious Diseases, Department of Internal Medicine, All India Institute of Medical Sciences, Jodhpur, India; 3 Department of Otolaryngology, All India Institute of Medical Sciences, Jodhpur, India; 4 Department of Microbiology, All India Institute of Medical Sciences, Jodhpur, India; 5 Department of Community Medicine & Family Medicine, All India Institute of Medical Sciences, Jodhpur, India; 6 Department of Pulmonary Medicine, All India Institute of Medical Sciences, Jodhpur, India

**Keywords:** COVID-19, Diabetes Mellitus, Invasive Mucormycosis, Risk Factors, Systemic Corticosteroids

## Abstract

**Background and Purpose:**

Rapid surge of invasive mucormycosis has surprised the Indian healthcare system amidst the coronavirus disease-19 (COVID-19) pandemic. Hence, there is an urgent need to find the risk factors for the sudden rise in cases of invasive mucormycosis among COVID-19 patients. This study aimed to find crucial risk factors for the sudden surge of invasive mucormycosis in India

**Materials and Methods:**

This case-control study included 77 cases of COVID-19 associated mucormycosis (CAM) who matched the controls (45 controls) in terms of age , gender, and COVID-19 disease severity. The control group included subjects that matched controls without mucormycosis confirmed by reverse transcription-polymerase chain reaction at our tertiary care center during April-May 2021. Probable predisposing factors, such as duration of diabetes mellitus (DM), history of recent hospitalization, duration of hospital stay, mode of the received oxygen supplementation, and use of steroids, zinc, vitamin c, and any other specific drugs were collected and compared between the two groups. Moreover, the laboratory parameters, like glycated hemoglobin (HbA1c), highly sensitive C-reactive protein (hs-CRP), and erythrocyte sedimentation rate (ESR) were analyzed to find out the significant association with CAM

**Results:**

DM (Odds ratio=7.7, 95% CI 3.30-18.12; *P*=<0.0001) and high glycated hemoglobin level (HbA1c>7.5 gm %) (odds ratio=6.2, 95% CI 1.4-26.7; *P*=0.014) were significant risk factors for the development of invasive mucormycosis among the COVID-19 cases. A higher number of mild COVID-19 cases developed CAM, compared to the moderate to severe cases (59.7% vs 40.3%). Use of systemic corticosteroids (odd ratio=5 with 95% CI 1.5-16.9; *P*=0.007) was found to be a risk factor for invasive mucormycosis only in mild COVID-19 cases. Use of oxygen, zinc, and vitamin C supplementation, and proprietary medicine did not lead to a significant risk of invasive mucormycosis in cases, compared to controls. Cases with invasive mucormycosis had a higher level of inflammatory markers (hs-CRP and ESR, *P*=<0.001 and 0.002, respectively), compared to the controls.

**Conclusion:**

Uncontrolled and new-onset DM and the use of systemic corticosteroids in mild cases were significantly associated with a higher risk of invasive mucormycosis in COVID-19 cases. There should be a strong recommendation against the use of systemic corticosteroids in mild COVID-19 cases

## Introduction

Coronavirus oronavirus disease 2019 (COVID-19) pandemic continues to be a major health problem worldwide. The second wave of COVID-19 has affected India substantially since its beginning in March 2021, accounting for approximately 26 million cases with 0.33 million mortalities as reported by the end of May 2021 [ 1 ]. During the early stages of the pandemic, bacterial infections were more common and only less than 1% of the secondary infections were fungal [ 2 ]. However, recently, during the second wave, reports of rising systemic fungal infections have raised a lot of concern [ 2 ]. Particularly, the recent unprecedented surge of COVID-19-associated invasive mucormycosis in India has attracted worldwide attention [ 3 , 4 ]. 

The incidence rate of mucormycosis has always been much higher in India, which is approximately 70 times more than the global average, even since the pre-COVID-19 era. This can be attributed to the fact that India is considered the diabetic capital of the world [ 5 , 6 ]. Invasive mucormycosis is considered a rare and fatal disease caused by *Mucorales* spp. of the phylum Zygomycota [ 7 ]. However, during the second wave of COVID-19, India witnessed a sudden rise of invasive mucormycosis cases as a red alert among the affected as well as the recovered patients of COVID-19 [ 8 - 19 ]. This condition is referred to as COVID-19 associated mucormycosis (CAM) in the literature [ 21 ].

More than one year has passed since the onset of this pandemic; nevertheless, its definitive treatment remains controversial. Results of the UK RECOVERY trial have proven the utility of steroids in improving outcomes in moderate to severe COVID-19 cases [ 10 ]. However, inadvertent use of steroids even in the mild COVID-19 cases was observed during the second wave of the pandemic worldwide. Immune dysregulation caused by corticosteroid use and the SARS-CoV-2 virus per se along with a high number of uncontrolled and undiagnosed type 2 diabetes mellitus (DM) patients in India, rendered a fertile ground for the development of invasive fungal infections, such as aspergillosis and mucormycosis [ 8 , 11 ]. 

Along with the above-mentioned factors, there were speculations about other risk factors contributing to the scenario, such as the use of industrial oxygen, zinc supplementation, steam inhalation, and unhygienic mask among COVID-19 patients. Therefore, this case-control study aimed to analyze the risk factors for the development of CAM among hospitalized patients with invasive mucormycosis in our tertiary center. Such a study will help to identify the high-risk groups for the development of CAM.

## Materials and Methods

This case-control study was conducted in the patients hospitalized with invasive mucormycosis at our tertiary care center in western India between April-May 2021. The cases and controls were selected from patients who were positive for COVID-19 by real-time reverse transcriptase-polymerase chain reaction (RT-PCR) with matched disease duration. The inclusion criteria for cases were admission with clinical and radiological suspicion of invasive rhino-sino-orbital mucormycosis and being microbiologically positive for *Mucorales* spp. 

Microscopically intra-operative or post-operative tissue samples showing broad aseptate or sparsely septate hyphae with obtuse angled branching were provisionally identified to be of *Mucorales* class in KOH mount or by histopathologic examination. Such samples were also subjected to fungal culture on Sabouraud Dextrose Agar (HiMedia, Mumbai) for confirmation. The cases and controls matched in terms of age, gender, and COVID-19 disease severity. The controls were selected from COVID-19 cases confirmed by RT-PCR who were either hospitalized or in home isolation with no clinical suspicion of invasive mucormycosis. The informed consent was taken from all study participants. The study was approved by the institutional Ethics Committee (reference number: AIIMS/IEC/2021/3655).

Demographical data of the subjects were collected, and the severity of COVID-19 was evaluated according to the guidelines issued by World Health Organisation on May 27, 2020 [ 22 ]. Symptomatic patients without evidence of viral pneumonia and hypoxemia (oxygen saturation by pulse oximetry [SpO_2_]: ≥94% on room air) were categorized as mild diseases. Patients with clinical signs of viral pneumonia and SpO_2_≥90% on room air were categorized as moderate cases. Moreover, cases with viral pneumonia plus one of the conditions of the respiratory rate of > 30 breaths/min and SpO_2_ < 90% on room air were categorized as severe cases.

Data were collected about the detailed history of the patients regarding the risk factors of invasive mucormycosis, like duration of DM, recent hospitalization, duration of hospital stay, mode of received oxygen supplementation, and use of steroids, tocilizumab, zinc, vitamin c, and any other specific medications. Steroid dosage was calculated as methylprednisolone (MPS) equivalent (32 mg MPS=6 mg Dexamethasone=40 mg Prednisolone) [ 23 ]. In addition, information about the hand hygiene practices and type of masks used in the last two months was collected. Moreover, blood samples were collected for relevant biochemical analysis, including glycated hemoglobin (HbA1c), highly sensitive C-reactive protein (HsCRP), and erythrocyte sedimentation rate (ESR), such as the routine workups.

It must be mentioned that the statistical analysis was performed in SPSS software (version 20.0, Armonk, NYN: IBM Corp). Continuous variables were expressed in mean±SD and categorical variables in number (percentage). Independent student’s t-test and Chi-square test were used to calculate the p-value for continuous data and categorical variables, respectively. The odd ratio was calculated for risk factors of invasive mucormycosis in COVID-19. It should be mentioned that a p-value of less than 0.05 was considered statistically significant. 

## Results

In total, 77 cases of invasive CAM were evaluated in this case-control study. The cases matched controls (45 controls) without invasive mucormycosis in terms of age, gender, and COVID-19 disease severity. Demographic characteristics of the subjects and presenting clinical features of both cases and controls were analyzed as shown in Table 1.

**Table 1:** Demographic characters and clinical features of COVID-19 associated mucormycosis

Variables	Cases (n=77)	Control (n=45)	*P-value*
Male (%)`	52 (67.5%)	33 (73.3%)	0.5
Age in years (Mean±SD)	46.8±13.1	46.6±15.2	0.34
Residence			
Rural (%)	29 (37.7%)	18 (40%)	1.0
Urban (%)	45 (58.3%)	27 (60%)	
History of working in farm (%)	20 (25.9%)	3 (6.6%)	0.003
COVID-19 disease severity (%)			
Mild disease	46 (59.7%)	24 (53.3%)	0.77
Moderate disease	22 (28.8%)	16 (35.6%)	
Severe disease	9 (11.7%)	5 (11.1%)	
Duration of COVID-19 illness (Mean±SD) (Days)	16.5±4.6	14.8±5.3	0.065
Presenting clinical features of invasive mucormycosis			
Fever	72 (93.5%)		
Nasal congestion	71(92.2%)		
Hemifacial pain	69 (89.6%)		
Periorbital swelling	56 (72.7%)		
Toothache	34 (44.2%)		
Headache	24 (31.2%)		
Local pain and tenderness	17 (22.1%)		
Loss of Vision	7 (9.1%)		
Brown/black discharge from nose	6 (7.8%)		

Comparison of the two groups revealed no significant differences between cases and controls in terms of age, gender, or location. However, regarding occupation, CAM was more prevalent among the people with a recent history of working in farms (*P*=0.003). Moreover, the incidence rate of CAM was found to be higher in patients with mild COVID-19 diseases, compared to those with moderate and severe disease (59.7% vs. 40.3%). 

Most of the mild cases were managed in home isolation and quarantine facilities (85%) and did not require hospitalization. The median duration of the onset of CAM after COVID-19 was 12 days (ranging from 2 to 27 days). All the CAM cases presented with rhino-sino-orbital mucormycosis with six (7.8%) patients having cerebral extension with invasion into cavernous sinus (ROCM). Clinically, the maximum number of patients with CAM presented with fever (93.5%), nasal congestion (92.2%), hemifacial pain (89.6%) followed by periorbital swelling (72.7%), dental pain (44.2%), and headache (31.2%). Only a few patients had extensive mucormycosis leading to loss of vision (9.1%). Furthermore, only 7.8% of patients presented with brown or blackish discharge from the nose.

Invasive mucormycosis was more prevalent in patients with DM and was a significant risk factor for invasive mucormycosis among all COVID-19 cases (odd ratio=7.7, 95% CI: 3.30-18.12; *P*<0.0001), specifically mild COVID-19 cases (odd ratio=10.7, 95% CI: 3.2-35.2; *P*<0.0001) (Tables 2-3). New-onset DM conferred a significant high risk of invasive mucormycosis (odd ratio=18.7, 95% CI: 2.4-144.3; *P*<0.0001) (Table-2). Furthermore, 65.2% of the new-onset DM belonged to mild COVID-19 cases. Uncontrolled DM (HbA1c>7.5 gm%) was associated with a statistically significant higher risk of CAM (odds ratio=6.26, 95% CI: 1.4-26.7; *P*=0.014) (Table-2). The mean HbA1c levels were significantly higher in cases, compared to the controls (Table-2); however, no case of diabetic ketoacidosis was reported in this study.

**Table 2:** Assessment of the risk factors of COVID-19 associated mucormycosis.

Risk Factors	Cases (n=77)	Control (n=45)	*P-value*
Diabetes mellitus	53 (68.8%)	10 (22.2%)	<0.0001
Oxygen use	31 (40.3%)	21 (46.7%)	0.75
New onset diabetes mellitus	23 (28.9%)	1 (1.3%)	<0.0001
Steroids use	54 (70.1%)	24 (53.3%)	0.206
Zinc used	44 (57.1%)	41 (91.1%)	0.007
Vitamin C used	41 (53.2%)	41 (91.1%)	0.002
Proprietary medicine	13 (16.9%)	4 (8.9%)	0.12
Steam inhalation	45 (58.4%)	24 (53.3%)	0.7
HbA1c>7.5 %	35 (45.5%)	3 (6.6%)	0.014
HbA1c (Mean±SD) (in %)	9.68±2.4	7.99±2.03	0.034
Inflammatory Markers (Mean±SD)			
HsCRP (pg/ml)	93.9±63.2	35.1±29.6	<0.001
ESR (mm in 1 hr)	60.8±24.9	41.6±27.9	0.002
Ferritin (ng/ml)	608.2±469.1	857.7±650.8	0.11

*HbA1c: glycated hemoglobin, HsCRP: highly sensitive C-reactive protein, ESR: Erythrocyte sedimentation rate

**Table 3:** Risk factors assessment of COVID-19 associated mucormycosis in mild COVID-19 cases

Risk factors	Cases (n=46)	Control (n=24)	*P-value*
Male	30 (65.2%)	15 (62.5%)	0.82
History of work in farm	13 (28.3%)	1 (4.2%)	0.01
Diabetes mellitus	34 (73.9%)	5 (20.8%)	<0.0001
New onset diabetes mellitus	15 (32.6.%)	1 (4.2%)	0.006
Steroids use	23 (50%)	4 (16.7%)	0.007
Diabetes mellitus+steroids	17 (36.9%)	2 (8.3%)	0.01
Zinc use	22 (47.8%)	24 (100%)	0.03
Vit C use	21 (45.7%)	22 (91.7%)	0.01
Proprietary medicine	7 (15.2%)	4 (16.7%)	0.82
MPS equivalent dosage (Mean±SD) (mg)	28.1±25.5	18±10.1	0.216
Duration of steroids use (Days)	7.8±4.3	3.3±1.3	0.048
Inflammatory markers (Mean±SD)
HsCRP (pg/ml)	93.8±58.6	23.5±23.1	<0.001
ESR (mm/h)	68.9±24.8	26.6±15.7	<0.001
Ferritin (ng/ml)	566.2±447.8	308.8±79.8	0.07

*MPS: methylprednisolone, HbA1c: glycated hemoglobin, HsCRP: highly sensitive C-reactive protein, ESR: erythrocyte sedimentation rate

Overall, systemic corticosteroids use in COVID-19 patients was not a significant risk factor for developing CAM (odd ratio=1.62 with 95% CI 0.77-3.41; *P*=0.206) (Table 2). However, the use of systemic corticosteroids in mild cases was associated with a significantly higher risk of the development of CAM (odd ratio=5 with 95% CI: 1.5-16.9; *P*=0.007) (Table-3). Extended duration of corticosteroid use was found statistically significant in invasive mucormycosis patients with mild COVID-19 (Table 3).

The other factors, such as the use of oxygen support by the patient, zinc and vitamin C supplementation, proprietary medicine intake, or steam inhalation for management of mild COVID-19 cases did not pose any significant risk for the development of CAM (tables 2 and 3). Given the inflammatory markers, HsCRP and ESR were markedly elevated in the cases when compared with controls (tables 2 and 3).

## Discussion

Invasive mucormycosis is infrequent in the population and is considered an opportunistic infection. The common predisposing factors are DM, use of systemic corticosteroids or immunosuppressant drugs, hematological malignancies, neutropenia, and stem cell transplantation [ 24 ]. There were only 82 cases of invasive mucormycosis reported during the first wave of COVID-19 from India [ 25 ]. However, the burden of invasive mucormycosis with COVID-19 was found to be much higher during the second wave of the pandemic than the first wave in India [ 21 ].

The two common presentations of invasive mucormycosis in the presence of the above-mentioned predisposing factors are ROCM and pulmonary mucormycosis [ 24 , 26 ]. However, in the present study, it was found that the predominant presentation of CAM as rhino-sino-orbital mucormycosis was followed by ROCM. On the contrary, Hoenigl et al. in their analysis reported ROCM as the most common presentation of CAM [ 27 ]. We also studied the risk factors for the development of CAM in the patients who previously had RT-PCR-confirmed COVID-19. 

The mean duration of developing CAM in post-COVID-19 patients was found to be 12 days (ranging from 2 to 27 days) in the present study. This was in concordance with the findings of the study performed by Pakdel F et al. who found that the mean duration of the development of CAM after recent COVID-19 diagnosis was 12.6 days (range=0-42 days) [ 28 ]. This finding suggests the importance of considering the signs of the development of CAM in post-COVID patients, especially up to the second week after the diagnosis of COVID-19. The present study highlighted that SARS-CoV-2 infection and its related medications may act as risk factors for mucormycosis and emphasized the need to monitor high-risk COVID-19 patients [ 9 ]. 

Uncontrolled diabetes with or without ketoacidosis is the major underlying disease causing mucormycosis cases in various geographical areas as reported previously [ 8 , 9 ]. India is known as the diabetic capital of the world, where the diagnosed cases constitute only the tip of the iceberg, compared to the actual number of cases [ 29 , 30 ]. In the present study, it was found that the patients with uncontrolled diabetes with high HbA1c values (>7.5 gm%) were at a significant high risk for developing CAM, similar to other studies [ 11 , 25 , 31 ]. Along with the high burden of DM, previously undiagnosed or newly diagnosed DM cases increased the risk of developing invasive mucormycosis following COVID-19 infections [ 25 , 32 ]. Few previous studies have found that people with diabetic ketoacidosis developed CAM promptly [ 25 , 33 , 34 ]. However, none of the cases of mucormycosis reported during our study period presented with diabetic ketoacidosis.

Corticosteroids have been used extensively and inadvertently for the treatment of COVID-19 to overcome the challenge of oxygen acquisition crisis for reducing the mortality rate in severely affected patients [ 15 ]. In a multicentre study performed in India, inappropriate corticosteroid use was noted in 63.3% of the patients [ 35 ]. In reported cases of CAM, significant percentages of cases (as high as 85.7%) were treated with steroids [ 11 , 24 , 28 , 35 ]. In this case-control study, the use of systemic corticosteroids was not found to be associated with the increased risk of invasive mucormycosis overall. 

Corticosteroid use in COVID-19 cases is known to act as a double-edged sword. They contribute to increased blood glucose levels by enhancing hepatic lipolysis, proteolysis, and glycogenolysis as well as by causing insulin resistance [ 36 ]. In diabetic patients, both hyperglycemia and insulin deficiency decrease the release of protective cytokines, which further reduces the macrophage activity and allows the pathogens to invade the immune system [ 16 ]. The use of systemic corticosteroids further suppresses the host immune system and precipitates hyperglycaemia, adding to the risk of secondary infections [ 37 ]. 

In the present study, it was found that the mild COVID-19 cases were at increased risk of developing CAM, compared to those having moderate and severe COVID-19 disease. Moreover, among the mild cases, uncontrolled and newly diagnosed diabetes and the use of corticosteroid and zinc supplementation led to the high risk of developing CAM. 

Most of the moderate to severe cases were managed at hospitals with judicious use of corticosteroids as per the standard treatment guidelines issued by the government of India with strict glycemic control. On the contrary, most of the mild cases were in home isolation and took conservative treatments, like antipyretics, zinc, and vitamin C supplementation. However, most of the mild cases had a history of steroid use due to its prescription by local medical practitioners even for mild symptoms. 

As per the UK recovery trial, the use of systemic corticosteroids showed mortality benefit in the COVID-19 patients requiring mechanical ventilation. However, no added benefits were obtained in the patients not requiring oxygen support [ 10 ]. Despite this fact, many local practitioners were seen prescribing corticosteroid even for the mild COVID-19 patients managed by home isolation during the second wave of COVID-19. Such irrational use of systemic corticosteroids as over-the-counter medications significantly increased the risk of CAM in mild COVID-19 cases as observed in this study. 

Working on the farm was found to be associated with higher exposure to fungal spores and increased risk of invasive mucormycosis. This disease was first diagnosed in farmworkers in China [ 38 ]. However, no such study has been performed so far to establish this association due to the rarity of this disease. In this research, it was found that the recent history of working on the farm was associated with an increased risk of mucormycosis.

The COVID-19 disease is associated with prolonged hyper inflammation, delayed release of cytokines, such as interferon-gamma, interleukin-1, interleukin-6, and tumor necrosis factor as well as decreased CD4 and CD8 cell count [ 12 , 39 ]. This defective immune response further led to ‘cytokine storm’ and multi-organ dysfunction, thereby suppressing the immune system and precipitating the risk of secondary bacterial and fungal infection [ 39 ]. The HsCRP and ESR are both important surrogate markers of inflammation and also correlated with disease severity in COVID-19. Furthermore, they were found to be associated with an increased risk of developing CAM in this study and other studies as well [ 40 , 41 ].

Analysis of 80 published cases from 18 countries has shown that uncontrolled diabetes and the use of systemic corticosteroids in the management of COVID-19 patients were the major predisposing factors for the development of CAM [ 27 ]. The triad of COVID-19, DM, and inadvertent use of systemic corticosteroids, especially in mild cases, may have been associated with an increased incidence rate of CAM in such a short span of time. The epithelial cell damage caused by acute respiratory distress syndrome in COVID-19 patients expose the extracellular matrix proteins, like laminin or collagen, and facilitate the attachment of inhaled or ingested fungal spores [ 42 ]. Nevertheless, the fungal spores of the Mucorales group are not known to interact with the intact epithelial cell layer.

The fungal hyphae of this group of pathogens possess a CotH (spore coating protein) receptor, which can facilitate their binding to the endothelial glucose-related protein78 (GRP78) receptor. This will further activate the platelet-derived growth factor receptor pathway leading to the endothelial cell invasion and injury [ 42 ]. The GRP78 receptor present on the endothelial cells has shown specificity for binding only to the Mucorales group, but not to other fungi, like Candida spp. or Aspergillus spp. [ 42 ]. Increased intracellular glucose and iron concentration can further upregulate the GRP78 receptor and enhance the risk of host cell invasion in mucormycosis cases [ 43 , 44 ]. It has been previously demonstrated that the expression of the GRP78 receptor was higher among the COVID-19 patients, compared to the non-COVID control group, which proves its pathogenic role in the development of CAM [ 45 ].

Oxygen therapy is the only proven treatment of COVID-19. Queries have also arisen that the use of supplemental oxygen allows the growth of the fungus in nasal mucosa; however, no studies are available to support this idea. In the present study, no significant risk of CAM was found among those patients who received any form of oxygen supplementation as the hospital- or home-based respiratory support. 

Zinc supplementation was used by almost every patient as an immune-booster against the COVID-19. It should be noted that zinc is an important element needed for fungal growth. Results of a recent in vitro study performed by Leonardelli et al. demonstrated the role of zinc chelation as an anti-fungal treatment in mucormycosis [ 35 ]. Therefore, zinc was proposed as a cause of this fungal epidemic in India. However, zinc and vitamin C use in COVID-19 treatment was not associated with an increased risk of invasive mucormycosis in this study. Most of the controls were also using zinc and vitamin C supplementation as a part of the standard kit issued by the government in mild cases. 

The main limitation of the study was the low number of controls and mere focus on the analysis of risk factors. The data regarding the extent of disease, high-resolution computer tomography chest score, response of treatment, and outcome are under study.

## Conclusion

Use of systemic corticosteroids should only be limited to moderate and severe COVID-19 patients in need of respiratory support; accordingly, they should not be used in mild cases as over-the-counter medications. The indiscriminate use of systemic corticosteroids with the high burden of undiagnosed cases of DM could be the probable explanation for this fungal epidemic in India. Immune dysregulation related to COVID-19 further complicated the situation.

## Acknowledgments

The authors would like to express their gratitude to Dr. Mahesh Soni’, Junior Resident, Department of Internal Medicine for his contribution to this research project.

## Authors’ contribution

S.K., D.K., and M.K.G. conceived the study. D.K., S.S., and S.K. collected the data. M.K.G., A.G., and D.K. performed the statistical analysis. M.K.G., D.S.M., and S.S. prepared the manuscript. All authors read and approved the final manuscript.

## Conflicts of interest 

The authors declare that there were no conflicts of interest in this study.

## Financial disclosure

No financial interests related to the material of this manuscript have been declared.

## Ethical Considerations

This study was approved by the Institutional Ethical Committee (reference number: AIIMS/IEC/2021/3655).
